# Antiviral Therapeutic Approaches for SARS-CoV-2 Infection: A Systematic Review

**DOI:** 10.3390/ph14080736

**Published:** 2021-07-28

**Authors:** Victoria Gil Martínez, Ana Avedillo Salas, Sonia Santander Ballestín

**Affiliations:** Department of Pharmacology, Physiology and Legal and Forensic Medicine, Faculty of Medicine, University of Zaragoza, 50009 Zaragoza, Spain; victoriiagil@gmail.com (V.G.M.); aave60@hotmail.com (A.A.S.)

**Keywords:** SARS-CoV-2, COVID-19, treatment, antiviral, remdesivir, lopinavir/ritonavir, favipiravir, ribavirin, umifenovir, arbidol, efficacy, safety

## Abstract

Due to the lack of an etiologic treatment for SARS-CoV-2 and the difficulties involved in developing new drugs, some drugs already approved for other diseases or with efficacy against SARS and MERS, have been used in patients with COVID-19. This systematic review aims to summarize evidence on the efficacy and safety of five antivirals applied to patients with COVID-19, that have proven to be effective either in vitro studies or in studies on SARS-CoV and MERS.; An intensive search of different databases (Pub Med, WoS, MEDLINE and Cochrane COVID-19 Study Register) has been carried out until the end of April 2021. This systematic review has been conducted according to the PRISMA statement. From each of the included studies, the characteristics of the intervention and comparison groups, demographic data and results were extracted independently; Remdesivir is well tolerated and helps to accelerate clinical improvement but is ineffective in reducing mortality. Favipiravir is safe and shows promising results regarding symptom resolution but does not improve viral clearance. The use of lopinavir/ritonavir has been associated with an increased risk of gastrointestinal adverse events and it has not proven to be effective. No significant differences were observed between patients treated with ribavirin or umifenovir and their respective control groups; Remdesivir and favipiravir are well tolerated and effective in accelerating clinical improvement. This systematic review does not support the use of lopinavir/ritonavir, ribavirin and umifenovir in hospitalized patients with COVID-19.

## 1. Introduction

### 1.1. Background

Members of the family Coronaviridae are widely spread among mammals, usually triggering respiratory infections [1]. Until 2002 it was assumed that these viruses were relatively mild human pathogens, responsible for 15% to 25% of common colds [2].

However, this perception changed in 2002 as a result of an outbreak of cases in China with severe respiratory symptoms, currently known as Severe Acute Respiratory Syndrome (SARS) [3]. A similar situation occurred in the countries of the Arabian Peninsula in 2012, with an outbreak now known as Middle East Respiratory Syndrome (MERS) [4,5].

The latest coronavirus with pathogenicity for humans that has been identified to date is SARS-CoV-2 [6]. At the end of December 2019, the headquarters of the World Health Organization (WHO) in China were informed of the presence of cases of pneumonia of unknown cause detected in the city of Wuhan [7]. Given its rapid expansion throughout the planet, the WHO confirmed on March 11, 2020 the existence of a pandemic [8].

SARS-CoV-2 is responsible for the disease known as COVID-19, whose dominant route of transmission is respiratory [9]. Its incubation period ranges between 2 and 11 days [10] and patients usually present the following symptoms: cough, dyspnoea, fatigue, fever, and sore throat. In severe cases, patients can develop pneumonia, acute respiratory distress syndrome (ARDS) or multiple organ failure [11].

### 1.2. SARS-CoV-2 Molecular Structure and Pathogenesis

**Figure 1:**
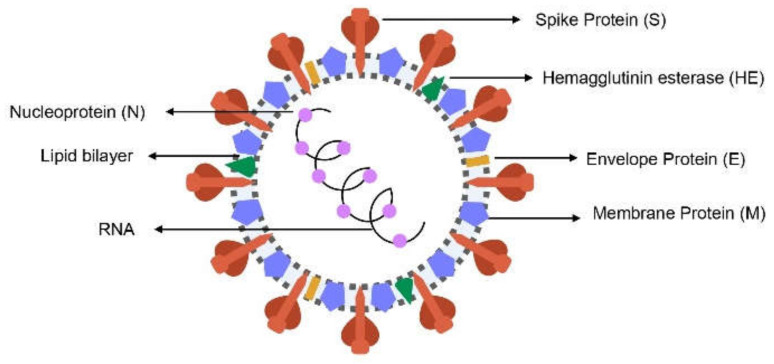
Representation of the structural proteins of SARS-CoV-2.

SARS-CoV-2 is a positive-sense single-stranded RNA virus (+ssRNA) [12] and encodes four structural proteins: nucleocapsid protein (N), spike protein (S), membrane protein (M) and envelope protein (E), as well as other non-structural proteins (nsp) (Figure 1) [13]. More specifically, the spike protein is a glycoprotein that forms homotrimers protruding from the viral surface [14] and facilitates the binding of the virus to the host cell receptor [15].

**Figure 2:**
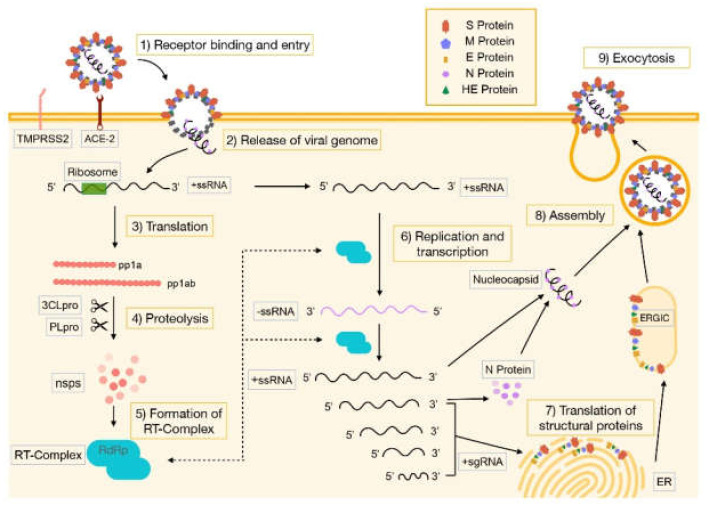
SARS-CoV-2 replication mechanism. ACE-2: angiotensin-converting enzyme 2, ER: endoplasmic reticulum, ERGIC: endoplasmic reticulum-Golgi intermediate compartment, nsps: non-structural proteins, PLpro: papain-like protease, Pp1a and pp1ab: polyproteins with 440–500 kDa and 740–180 kDa respectively, RdRp: RNA-dependent RNA polymerase, RT-Complex: replicase-transcriptase complex, + sgRNA: positive-sense subgenomic RNA, + ssRNA: full-length positive-sense single-stranded RNA, -ssRNA: full-length negative-sense single-stranded RNA, TMPRSS2: transmembrane protease serine 2, 3CLpro: chymotrypsin-like cystine protease.

The entry of SARS-CoV-2 into the host cell occurs thanks to the binding of the viral spike (S) proteins to its host target cell receptor, which is the angiotensin-2 converting enzyme (ACE-2) [16]. This peptidase is present in a large number of cells in our body, but it is mainly expressed on lung alveolar epithelial cells and enterocytes of the small intestine [17]. The activity of a host cell surface protease, called transmembrane protease serine 2 (TMPRSS2) is essential for this process to take place (Figure 2) [18,19,20,21,22].

### 1.3. Immune Response and Clinical Features

As the virus replicates inside the host cell, the production of type I interferon (IFN) begins. It will interact with other cells of the immune system such as macrophages and neutrophils, considered major sources of pro-inflammatory cytokines and chemokines such as IL-1β, IFNγ, tumor necrosis factor alpha (TNFα), inducible protein-10 (IP-10) and monocyte chemoattractant protein- 1 (MCP-1), which can lead to the activation of T-helper-1 cells (Th1) [23]. In addition, IL-17, produced by Th17 cells, recruits monocytes and neutrophils to the site of infection, boosting inflammation. Finally, some anti-inflammatory cytokines such as IL-4 and IL-10 are also released, in an attempt to counteract this inflammatory process [23]. 

The most frequent symptoms are fever, cough, asthenia, dyspnoea, myalgia or arthralgia, ageusia and anosmia [11,24]. Other less frequent ones are a productive cough, sore throat, headache, nausea and vomiting, nasal congestion, diarrhoea, haemoptysis, conjunctivitis and skin manifestations [11,24]. 

If the immune response is effective, approximately 80% of infected patients have a mild or even an asymptomatic clinical course [25]. However, the remaining 20% progress to more serious stages leading to admission to intensive care unit (ICU), ARDS, septic shock, or multiple organ failure [25]. Likewise, in patients with an exacerbated inflammatory response, a dysfunctional cascade of inflammatory thrombosis is triggered, which leads to a state of hypercoagulability, responsible for thrombosis at micro and macrovascular level [26].

### 1.4. Therapeutic Possibilities

Up to the present day we lack an etiological treatment against COVID-19, which is why the therapeutic strategies used are being oriented in three main directions [27].

One way is trying to reduce or eliminate the SARS-CoV-2 viral load using molecules capable of interfering with viral replication. Another way is using immune therapies that allow regulating the inflammatory response and finally, the other therapeutic strategy used is symptomatic treatment, for example, providing oxygen [27]. Antiviral drugs are considered useful in the early stages of the disease when active viral replication is still prevalent. However, in later stages a pro-inflammatory process stands out, where the use of immunomodulatory agents is being evaluated [27].

Some of the antivirals used against SARS-CoV-2, among other non-antiviral molecules are shown in Table 1 [23,28,29,30,31,32,33,34,35].

#### Vaccination

Since the beginning of the pandemic, a major scientific effort has been made to develop a vaccine that will make it possible to immunize the population and thus reduce the incidence of this virus. As of 14 July 2021, a total of 3,400,884,367 vaccine doses have been administered around the world [36]

Currently there are three main types of vaccines against SARS-CoV-2 virus. On the one hand, the messenger-RNA (mRNA) vaccines use manufactured nucleoside-modified, single stranded mRNA, that contain the genetic code for the synthesis of the SARS-CoV-2 S protein antigen [37,38]. Given the fragility of this molecule, it enters the human cells encapsulated by lipid nanoparticles [37]. Once it is injected, the human body cells start synthetising the S protein, which induce a humoral and T-cell mediated immune response [37,38]

It should be noted that this type of vaccine is very vulnerable at room temperature, which is why it must be stored at −70 °C or −20 °C. This can be a problem in developing countries, where the continued supply of electricity to large industrial freezers is unreliable [39].

Nowadays there are two COVID-19 mRNA vaccines approved for market: mRNA-1273 developed by Moderna and BNT162b2 developed by Pfizer-BioNTech [39]. On the other hand, we have the adenovirus vector-based vaccines, which use a modified human (Ad5 and Ad26) or chimpanzee adenovirus (ChAdOx1) to deliver the genetic code for the SARS-CoV-2 Spike protein antigen [37,40]. Once it enters the human body cells, the delivered genetic material escapes from the vectors and will be used to synthetize the S-protein antigen that will afterwards induce an immune response [37,40]. Currently, there are four vaccines with this technology approved for use in humans: Oxford–AstraZeneca vaccine, Sputnik-V vaccine, Johnson and Johnson vaccine and AD5-nCoV (Convidecia) vaccine [37].

We can also find inactivated coronavirus vaccines, created by using radiation, heat or chemical stress [41]. This type of vaccines contains antigens that once injected into the body activate the immune system [38]. Their downside is that they usually need additional adjuvants [41]. One example of an authorized inactivated coronavirus vaccine for human use is the Sinopharm vaccine [37].

### 1.5. Justification and Aim

Given the urgent need to reduce the cost, time and risks derived from the development of new medicines, drugs already approved for other indications have been ‘’repurposed’’ and used to treat COVID-19 patients.

Nowadays, not too many studies regarding the use of antiviral drugs on patients with SARS-CoV-2 infection have been carried out. The present study aims to summarize evidence on the efficacy and safety of five antivirals applied to patients with COVID-19, that have proven to be effective either in in vitro studies or in studies on SARS-CoV and MERS [42,43].

## 2. Methods

### 2.1. Search Strategy

The search was made using PubMed, Scopus, Web of Science, MEDLINE, and Cochrane COVID-19 Study Register, which also includes trials published in ClinicalTrials.gov-COVID-19 subset and WHO International Clinical Trials Registry Platform (ICTRP).

The studies have been identified by combining the name “coronavirus”, “COVID-19” and “SARS-CoV-2” with the following keywords: lopinavir/ritonavir, remdesivir, favipiravir, umifenovir, ribavirin or their respective trade names. MeSH terms (Medical Subject Headings) and the Boolean operators “AND’’ and ‘’OR’’ have also been used in the search.

**Figure 3:**
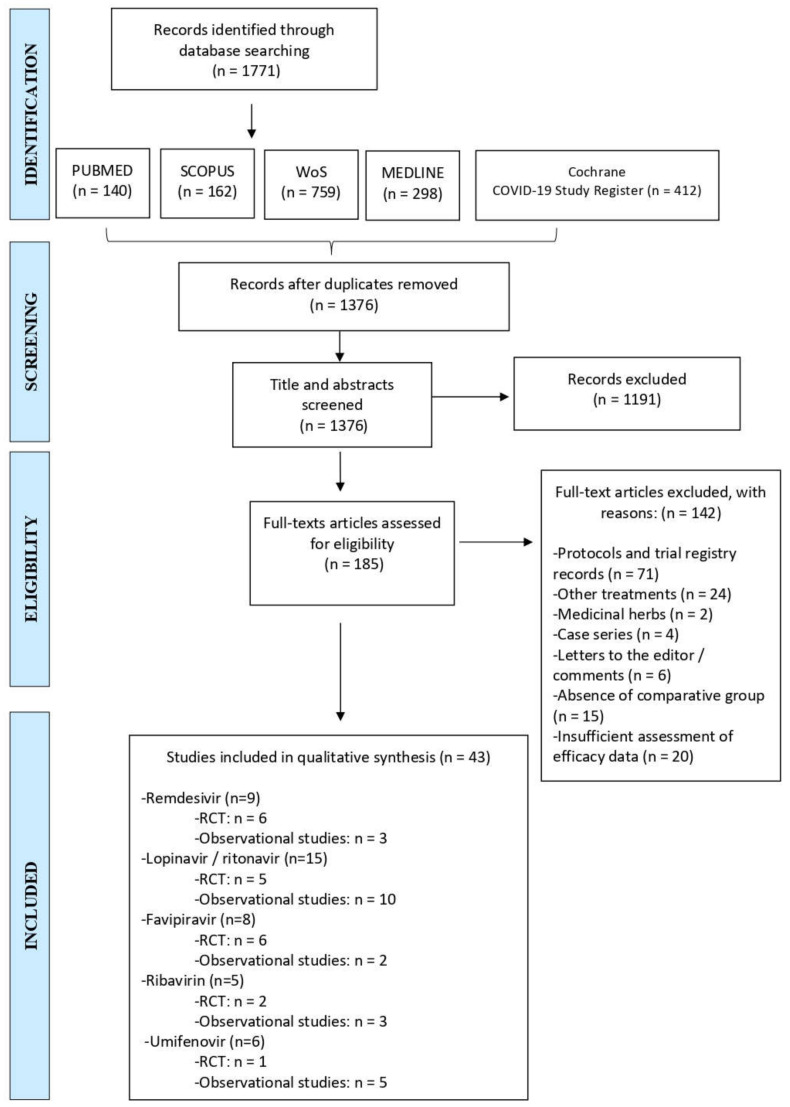
PRISMA 2009 Flow diagram illustrating the study selection process [44]. RCT: randomized controlled trial, WoS: Web of Science.

The study was conducted according to the PRISMA statement [44]. Articles published between January 2020 and April 2021 were retrieved. After removing duplicates, titles and abstracts were screened excluding those that did not meet the inclusion criteria. The remaining records have then been assessed for eligibility by careful review of their full texts. A flow chart illustrating the study selection process is shown in Figure 3.

### 2.2. Inclusion Criteria

Regarding type of studies, both randomized controlled clinical trials (RCTs) and observational studies were included. The rest of the inclusion criteria have been proposed according to the PICO algorithm (Table 2).

### 2.3. Exclusion Criteria

The proposed exclusion criteria for this systematic review have been: (a) studies with insufficient data, (b) in vitro, in silico, in vivo animal studies, (c) comments, expert opinions, case reports, letters to the editor, reviews, protocols and trial registry records, (d) studies including patients with other coronaviruses, SARS, or MERS, (e) studies that do not include as an intervention at least one of the five antivirals evaluated in this systematic review, (f) studies using medicinal plants.

### 2.4. Data Collection and Analysis

The following information has been extracted from each of the included studies: (a) clinical trial registration number, (b) author, (c) publication date, (d) trial design, (f) participant characteristics (severity of the disease), (g) country, (h) interventions carried out with their respective dosage regimen, (i) comparison group dosage regimen (i) number of participants recruited/allocated/evaluated, (j) age and gender of participants, (k) outcomes.

## 3. Results

### 3.1. Remdesivir

#### 3.1.1. Efficacy

We included nine studies about remdesivir, which is the first FDA-approved drug for COVID-19 [45]. Six of them are randomized controlled trials (RCTs) and three are observational studies (Table 3).

One of the RCTs carried out in multiple centres in the USA, Europe and Asia simultaneously, compared a 10-day course of remdesivir and a 5-day course with standard of care [46]. Patients with moderate COVID-19 pneumonia receiving a 5-day remdesivir therapy presented significantly higher odds of having a better clinical status distribution on day 11 than those receiving standard of care (OR = 1.65; 95% CI = 1.09–2.48; *p* = 0.02) [46]. In contrast, patients randomized to a 10-day regimen of remdesivir did not show a statistically significant difference compared with standard of care (*p* = 0.18) [46]. Other outcomes such as length of hospital stay, all-cause mortality at day 28 or time to recovery did not show significant differences (*p* > 0.05) [46].

Goldman et al. compared a 5-day course of remdesivir with a 10-day course in patients with severe COVID-19. Despite an apparent trend towards better outcomes in patients treated with remdesivir for 5 days than those receiving it for 10 days, the differences were not statistically significant. After adjusting for baseline imbalances in severity disease, similar results were obtained between both groups in terms of clinical status at day 14 (*p* = 0.14), length of hospital stay, discharge rate, time to recovery, and mortality [47]. However, these results cannot be extrapolated to critically ill patients receiving mechanical ventilation, as few of the patients included in this trial were already receiving mechanical ventilation prior to initiating treatment with remdesivir [47].

The first phase of the “Adaptive COVID-19 treatment trial or ATT-1” constitutes a RCT where 541 patients allocated to remdesivir therapy were compared with 521 patients randomized to placebo [48]. The data obtained show that patients receiving remdesivir had a shorter time to recovery than those receiving placebo (10 days vs 15 days respectively, with a RR for recovery = 1.29; 95% CI = 1.12–1.49; *p* <0.001) as well as higher odds of improvement in the eight-category ordinal scale on day 15, where “1” corresponds to not having any limitations and “8” means death (OR = 1.5; 95% CI = 1.2–1.9) [48]. 

Additional secondary end points that support the use of remdesivir are: a shorter time to improvement of one and of two categories on the 8-category ordinal scale, a shorter time to discharge, and a shorter length of hospital stay (12 days versus 17 days in the remdesivir and placebo groups respectively). Moreover, mortality by day 29 was 11.4% in the remdesivir group versus 15.2% in the placebo group (HR = 0.73; 95% CI = 0.52–1.03) [48].

Wang et al. recruited a total of 237 severe COVID-19 patients (158 randomized to remdesivir therapy and 78 to placebo) in China at the start of the pandemic [49]. Although not statistically significant, patients receiving remdesivir presented a shorter time to clinical improvement (21 days) compared to those in the placebo group (23 days) (HR = 1.23, 95% CI = 0.87–1.75) [49]. Time to clinical improvement was defined as the time in days from randomization until a two-level improvement on a six-category ordinal scale where 1 = discharge and 6 = death [49]. Other outcomes such as mortality or time to viral clearance (*p* = 0.0672) did not show a significant improvement in the intervention group either [49]. It is important to mention that this trial did not reach its target enrolment given the strict public health measures used in Wuhan. Consequently, it cannot be adequately assessed whether early treatment with remdesivir could have provided a clinical benefit [49].

These results are in line with those of the ‘’WHO Solidarity Trial Consortium”, in which several intervention arms were compared with standard of care. The use of remdesivir as monotherapy did not significantly reduce 28-day mortality (RR = 0.95; 95% CI = 0.81–1.11; *p* = 0.50) nor the percentage of patients requiring mechanical ventilation after randomization [50].

One of the observational studies included in this systematic review retrospectively compared 25 critically ill patients undergoing mechanical ventilation, who had received remdesivir with 26 patients treated with standard of care [51]. In this study, remdesivir was associated with a beneficial effect in terms of survival (OR = 3.506; 95% CI = 1.768–6.954; *p* <0.001) [42]. It should be mentioned that the general mortality recorded in this study was one of the highest reported in literature. However, the patients included were critically ill [42].

Olender et al. used data from a phase 3 remdesivir RCT and compared it with a longitudinal retrospective cohort of patients treated with standard of care [52]. By day 14, the proportion of recovered patients was significantly higher in the remdesivir cohort compared to the control group (OR = 2.03; 95% CI = 1.34–3.08; *p* < 0.001). In addition, remdesivir was associated with greater clinical improvement, assessed using a 7-category ordinal scale, as well as, with 62% lower odds of all-cause death compared with the standard of care cohort (OR = 0.38; 95% CI = 0.22–0.68; *p* = 0.001) [52]. 

In line with the results seen so far, there are also those observed in another retrospective study where a group of 99 patients treated with remdesivir was compared with 125 patients who had received supportive care [53]. Despite not reaching statistical significance, patients treated with remdesivir presented numerically lower all-cause in-hospital mortality (HR = 0.44; 95% CI = 0.16–1.23; *p* > 0.05). No significant differences could be found in terms of time to clinical recovery and time to discharge (RR = 1.26; 95% CI = 0.83–1.92; *p* > 0.05 and RR = 1.24; 95% CI = 0.81–1.90; *p* > 0.05, respectively) [53].

Finally, there is a double-blind RCT that has analysed the potential efficacy of remdesivir in combination with another drug. More specifically, it compares the use of remdesivir associated with barticinib versus remdesivir + placebo [54]. This study has shown that the combination treatment is superior to remdesivir alone in reducing recovery time (RR = 1.16; 95% CI = 1.01–1.32; *p* = 0.03) and in increasing improvement in clinical status by day 15 (OR = 1.3; 95% CI = 1.0–1.6) [54].

#### 3.1.2. Safety

The RCT by Spinner et al. showed that those randomized to a 10-day course of remdesivir had significantly higher rates of adverse events compared to those receiving standard of care (*p* = 0.02). Adverse events that were more common in the remdesivir group than in the standard of care group were: nausea, hypokalaemia, and headache. With regard to severe adverse events, they were numerically lower in both remdesivir intervention arms than in the control group, albeit not statistically significant [46].

Meanwhile, the study by Goldman et al. did not find significant differences regarding the rate of adverse events between patients randomized to a short course of remdesivir (5 days) and those allocated to a long course of remdesivir (10 days) [47].

Data from the ACTT-1 trial suggest that remdesivir treatment may have prevented the progression to more severe respiratory disease. This can be evidenced by the lower proportion of serious adverse events due to respiratory failure among patients in the remdesivir group as well as a lower incidence of new use of oxygen among patients who were not receiving oxygen at the time of recruitment (36% vs. 44%, in the remdesivir group and placebo group respectively) [48].

In the RCT proposed by Wang et al. remdesivir was well tolerated and no new safety concerns were identified. The overall proportion of patients with serious adverse events tended to be lower in remdesivir-treated patients (18%) than in placebo-treated patients (26%) [49].

Nevertheless, there was a higher rate of premature discontinuation of treatment in the intervention group (12%) compared to the placebo group (5%) because of adverse events such as gastrointestinal symptoms (anorexia, nausea and vomiting), increases in aminotransferase or bilirubin levels and worsening of cardiopulmonary status [49].

Another observational study found that the therapy with remdesivir did not increase liver test abnormalities and was not associated with a higher risk of acute kidney injury (HR = 1.10; 95% CI = 0.64–1.90; *p* > 0.05) [53].

In the case of the study that has compared the combination of remdesivir with barticinib versus remdesivir alone, those receiving dual therapy have presented significantly fewer serious adverse reactions (16% vs. 21%, respectively; *p* = 0.03) [54].

### 3.2. Lopinavir/Ritonavir

#### 3.2.1. Efficacy

Five RCTs and ten observational studies regarding lopinavir/ritonavir have been included (Table 4).

In an RCT carried out in China, 99 patients with severe COVID-19 randomized to lopinavir/ritonavir were compared with those allocated to standard of care. This study found that treatment with lopinavir/ritonavir was not associated with significant clinical improvement, mortality reduction or reduction in viral RNA detectability, since SARS-CoV-2 RNA was still detectable in 40.7% of the patients treated with lopinavir/ritonavir at the end of the study (day 28) [55]. It should be noted that the overall mortality in this trial was 22.1% [55], significantly higher than the percentages reported by initial descriptive studies (around 11% and 14.5%) [11,24], which would be explained by the fact that this study included severely ill patients [55].

The results of the RECOVERY trial carried out in the United Kingdom, which enrolled 5040 patients, indicate that compared to standard of care, lopinavir/ritonavir is not associated with significant reduction in mortality (RR = 1.03; 95% CI = 0.91–1.17; *p* = 0.60), in length of hospital stay, as well as in risk of progressing and needing invasive mechanical ventilation (RR = 1.09; 95% CI = 0.99–1.20; *p* = 0.092), which is why they conclude that lopinavir/ritonavir monotherapy is not an effective treatment for hospitalized COVID-19 patients [56].

In the RCT by Hung et al., the patients allocated to the triple therapy of lopinavir/ritonavir + ribavirin + IFN-ß1b were compared with those in the control group receiving lopinavir/ritonavir monotherapy. The results show that this triple therapy applied within 7 days of symptom onset is effective in suppressing SARS-CoV-2 shedding [57]. This positive clinical and viral response is also reflected in a significant shorter median duration of hospital stay (HR = 2.72; 95% CI = 1.2–6.13; *p* = 0.016), as well as in a faster resolution of symptoms assessed by the SOFA score (HR = 1.89; 95% CI = 1.03–3.49; *p* = 0.041) [57]. 

The DisCoVery trial is an RCT carried out in multiple French healthcare centres simultaneously, in which several lines of treatment have been investigated [58]. While in one of the intervention arms patients were randomized to lopinavir/ritonavir in monotherapy, in another arm the patients were allocated to a combination therapy of lopinavir/ritonavir + IFN-β1a. Both intervention arms were compared with a group of patients assigned to standard of care [58].

None of these investigational treatments resulted in significant clinical improvement at day 15 and at day 29, as measured on the WHO-7-point ordinal scale, where “1” implies the absence of activity limitations or need for hospitalization and “7” equals death [58].

In more detail, the odds for clinical status improvement at day 15 in the lopinavir/ritonavir monotherapy group versus the standard of care group was OR = 0.83 (95% CI = 0.55–1.26; *p* = 0.39), while at day 29 it was OR = 0.93 (95% CI = 0.62–1.41; *p* = 0.74) [58]. In the case of dual therapy (lopinavir/ritonavir+ IFN-β1a) compared to standard of care, the odds for clinical improvement at day 15 was OR = 0.69 (95% CI = 0.45–1.04; *p* = 0.08) and at day 29 it was OR = 0.76 (95% CI = 0.50–1.15; *p* = 0.19) [49]. Likewise, no statistically significant differences were identified in terms of mortality, oxygenation-free days, and ventilator-free days between patients who received lopinavir/ritonavir, either in monotherapy or associated with IFN-β1a, and patients in the control group [58].

These results are in line with those of the WHO Solidarity Trial Consortium, in which several lines of treatment were compared with standard of care internationally. One of them was lopinavir/ritonavir in monotherapy, but it failed to reduce mortality (RR = 1.00; 95% CI = 0.79–1.25; *p* = 0.97) and the percentage of patients requiring mechanical ventilation after randomization [50].

Regarding the observational studies included, Gao et al. have found no evidence that lopinavir/ritonavir monotherapy accelerates the remission of symptoms like fever (OR = 0.910; 95% CI = 0.802–1.033; *p* = 0.146), the time to viral clearance (OR = 1.018; 95% CI = 0.985–1.051; *p* = 0.285), nor prevents the progression to severe cases (OR = 1.163; 95% CI = 0.158–8.568; *p* = 0.882) [59].

Following this line of results, Grimaldi et al. compared a total of 57 patients receiving lopinavir/ritonavir with a cohort of 85 patients treated with standard of care [51]. Here, the treatment with lopinavir/ritonavir was associated with significant lower chance to be alive and extubated at day 28 (OR = 0.41, 95% CI = 0.20–0.83). Due to the lack of benefit, this study does not support the use of lopinavir/ritonavir in patients with moderate-severe COVID-19 [60].

The same occurs in the retrospective cohort study by Choi et al., where the median viral shedding duration was longer in patients treated with lopinavir/ritonavir (23 days) compared to those who only received standard of care (18 days) (*p* < 0.0001) [61].

On the contrary, Ye et al., observed that patients treated with lopinavir/ritonavir monotherapy accelerated time to viral clearance (*p* = 0.0219), as well as time to defervescence (*p* = 0.0364) when compared to standard of care [62].

Another study that failed to detect statistically significant differences in aspects such as treatment escalation (intubation, ECMO), ventilator free days, 14-day mortality and 28-day mortality and change in viral load between admission and day 7, was the retrospective study by Lecronier et al., where 20 patients treated with lopinavir/ritonavir were compared with 22 receiving standard of care [63].

Lu et al. studied the use of lopinavir/ritonavir monotherapy in paediatric patients with mild COVID-19. They could observe that the intervention group had a disadvantage compared with those receiving standard of care, in terms of time to viral clearance (HR = 5.33; 95% CI = 1.94–14.67; *p* = 0.001) and median duration of hospital stay (HR = 2.01; 95% CI = 1.24–3.29; *p* = 0.005) [64].

Other authors have tried to evaluate the differences in clinical efficacy between an early treatment (begin within 5 days of symptom onset) of lopinavir/ritonavir + hydroxychloroquine and a delayed treatment [65]. The results obtained do not show any statistically significant differences between groups regarding clinical improvement (*p* = 0.213) and 30-day mortality (*p* = 0.271) [65].

Lopinavir/ritonavir has also been compared with other antivirals. In a retrospective study carried out in China 36 patients who received lopinavir/ritonavir were compared with 16 patients treated with arbidol (trade name of umifenovir) [66]. Arbidol monotherapy was more effective than lopinavir/ritonavir, since all the patients who received arbidol presented an undetectable viral load on day 14 after admission, while only 55.9% of the patients treated with lopinavir/ritonavir (*p* < 0.01). Additionally, the duration of positive RNA test was shorter in the arbidol group (*p* < 0.01) [66].

Vernaz et al. propose in their retrospective single-centre study several intervention arms containing lopinavir/ritonavir. In one of them lopinavir/ritonavir monotherapy is compared with standard of care and no statistically significant differences can be found between both groups in terms of length of hospital stay (*p* = 0.319) or in-hospital mortality (*p* = 0.639) [67].

In another of the intervention arms, a group of patients treated with hydroxychloroquine + lopinavir/ritonavir was compared with a cohort that had received standard of care. This combination therapy failed to reduce mortality (*p* = 0.697) and it was significantly associated with an increase in length of stay (*p* < 0.001) [67].

Finally, the last observational study included in this systematic review coincides with the results proposed by Yan et al., since the group of patients treated with lopinavir/ritonavir had a significant shorter duration of viral shedding compared to the control group (*p* = 0.02). However, subgroup analysis revealed that this shorter duration was only statistically significant in those patients treated within 10 days from symptom onset (log-rank *p* < 0.0001), unlike those who received it eleven or more days later (log-rank *p* = 0.51) [68]. It is to be noted that the median duration of viral shedding, even in patients who received early administration of lopinavir/ritonavir was 19 days, demonstrating that lopinavir/ritonavir is not capable of completely inhibiting SARS-CoV-2 replication [68].

#### 3.2.2. Safety

Cao et al. conclude that gastrointestinal adverse events were more common in lopinavir/ritonavir group than in those who received standard of care. However, the number of patients with severe complications (acute renal failure and secondary infections) was higher in the control group. In total, 13 patients had to stop prematurely the use of lopinavir/ritonavir because of adverse events [55].

On the contrary, the DisCoVery trial recorded a significantly higher number of serious adverse events in those treated with lopinavir/ritonavir, either in monotherapy or associated with IFN-β1a. The most frequently reported ones were: acute respiratory failure (11%), acute kidney injury (8.2%), acute respiratory distress syndrome (8%), arrhythmia (7%), pulmonary embolism (5%) and sepsis (4%) [58].

Hung et al. reported no statistically significant differences between intervention and control group regarding adverse events (*p* > 0.05), being those side effects generally mild and self-limiting [57]. Ye et al. agree with these results and they not only do not detect liver toxicity, but they also observe an accelerated remission in white blood cell, lymphocyte and C-reactive protein abnormalities in those treated with lopinavir/ritonavir [62].

Grimaldi et al. registered a statistically significant higher incidence of cases of acute kidney injury (AKI) and need for renal replacement therapy (RRT) at day 28 in the lopinavir/ritonavir group compared to the control group (*p* = 0.002), which is why this study raises concerns about the safety profile of this antiviral [60].

In the study by Lu et al. the use of lopinavir/ritonavir in paediatric patients was associated with significantly higher adverse reactions compared to those who received conventional treatment, mainly because of a longer nasopharyngeal swab negativitation time (HR = 4.67; 95% CI = 1.35–16.11; *p* = 0.015) [64].

Finally, coinciding with Cao et al., a retrospective study also observed that the most frequent adverse events in patients treated with lopinavir/ritonavir were gastrointestinal symptoms including nausea, diarrhoea and elevated liver enzymes, in fact 8.1 % of the patients developed grade 2–3 gastrointestinal disorders and had to discontinue the treatment prematurely [55].

### 3.3. Favipiravir

#### 3.3.1. Efficacy

After literature search and careful review of full-text articles, a total of six RCTs and two observational studies on favipiravir have been included (Table 5).

One of the RCTs carried out in Japan, compared the early administration of favipiravir in patients with asymptomatic or mild COVID-19 with its late administration (starting on day 6 of study) [69]. Early favipiravir administration showed a trend towards earlier viral clearance, albeit not statistically significant (HR = 1.42; 95% CI = 0.76–2.62; *p* > 0.05) [69]. What was slightly significant was the reduction in time to defervescence (HR = 1.88; 95% CI = 0.81–4.35; *p* = 0.048) [69].

Another RCT was carried out in India and it compared the use of favipiravir in adults with mild to moderate COVID-19 with supportive care [70].

While a statistically significant difference in terms of time to cessation of viral shedding (HR = 1.367; 95% CI = 0.944–1.979; *p* = 0.098) and of time to hospital discharge (HR = 1.406; 95% CI = 0.974–2.030; *p* = 0.069) could not be reached, significant results were found regarding a reduction in time to clinical cure in those treated with favipiravir (HR = 1.749; 95% CI = 1.096–2.792; *p* = 0.019) [70]. This leads to the conclusion that early favipiravir administration may contribute to the reduction of symptoms and clinical signs in patients with mild to moderate COVID-19 [70].

The results obtained in another RCT carried out in Russia, in which 17 patients receiving favipiravir monotherapy were compared with 22 patients receiving standard of care, have been encouraging [71]. It should be noted that the standard of care consisted mainly of combinations of hydroxychloroquine, azithromycin, and lopinavir/ritonavir [71].

Favipiravir turned out to be more effective than the standard etiotropic therapy used as control group in various aspects such as: in reducing the time to defervescence, in having lower lactate levels in blood at the end of the treatment and in reducing the area of pulmonary parenchyma lesion according to the CT data (*p* < 0.05 respectively) [71].

In Egypt, the authors Dabbous et al. found in their RCT promising results regarding the use of favipiravir when compared to chloroquine. Although the differences were not statistically significant, those receiving favipiravir had a shorter duration of hospital stay (*p* = 0.06), lower mortality rates (*p* = 1.00) and none of them required mechanical ventilation (*p* = 0.118) [72].

In a phase II/III RCT, Ivashchenko et al. studied two possible favipiravir dosing regimens and compared each of them with a standard of care. In this study the patients were randomized 1:1:1 to receive either 1600 mg favipiravir, twice the first day, followed by 600 mg twice a day for 14 days, or 1800 mg favipiravir, twice the first day followed by 800 mg twice a day for 14 days or standard of care [73]. On both dosing regimens, the viral clearance rate by day 5 was twice as high as in the control group (*p* = 0.018) [73]. Likewise, both intervention arms normalized body temperature in less time than the control group (*p* = 0.007) [73].

In the last included RCT, three possible intervention arms were compared: favipiravir + tocilizumab; favipiravir monotherapy and tocilizumab monotherapy [74]. The simultaneal use of favipiravir with an IL-6 receptor antagonist proved to be superior than favipiravir monotherapy in improving pulmonary inflammation (HR = 2.66; 95% CI = 1.08–6.53; *p* = 0.019) and in reducing mortality [74]. The combination therapy was also associated with significant relieve of clinical symptoms (fever, cough, and dyspnoea) and normalization of blood routine [74].

Combinations of favipiravir with other drugs were studied in the following observational studies. Cai et al. proposed a comparative study in which the intervention group received favipiravir and IFN-α1b, while the control group received lopinavir/ritonavir associated with IFN-α1b. Those treated with favipiravir appeared to accelerate viral clearance (*p* < 0.001), as well as to improve chest CT inflammatory changes on day 14 after treatment (*p* = 0.004) [75].

In a retrospective study carried out in Turkey favipiravir was also compared with lopinavir/ritonavir, however, in this case both were simultaneously given with hydroxychloroquine [76]. On the one hand, those treated with lopinavir/ritonavir presented lower mortality rates (54.8%) than those treated with favipiravir (66.2%). However, this difference was not statistically significant (*p* = 0.237), which could be due to the small sample size of the study. On the other hand, favipiravir was superior to lopinavir/ritonavir in shortening the length of ICU stay (*p* = 0.01) [76].

#### 3.3.2. Safety

The most frequent adverse event recorded in 84.1% of the patients after favipiravir administration was hyperuricemia. Other reported adverse events were: elevation of serum triglycerides (11%) and serum alanine aminotransferase elevation (8.5%) [69].

These observed adverse events coincide with those of Udwadia et al., where most of them were mild and transient. Hyperuricemia and elevated liver enzymes were the most common ones detected in the favipiravir group. The reported gastrointestinal disorders were minimal and similar between intervention and supportive care groups [70].

In another RCT, it was found that, in terms of QTc prolongation, the use of favipiravir was safer than standard therapy, represented mostly by hydroxychloroquine and azithromycin. The rest of the recorded side effects were mild in form and associated with hepatotoxicity [71].

Following this line of results, the undesirable events reported by a study with two dosing regimens for favipiravir were mild to moderate in severity and consistent with those reported so far for this antiviral. In addition, those who received higher doses of favipiravir did not experience any increasing toxicity [73].

In one of the studies comparing the efficacy of favipiravir versus lopinavir/ritonavir, higher rates of adverse events in lopinavir/ritonavir group (55.56%) were noticed compared to favipiravir group (11.43%) (*p* < 0.001), being the most frequent one gastrointestinal disorders including diarrhoea, liver injury, nausea and vomiting [75]. On the contrary, Kocayiğit et al., found no significant differences between the group of patients treated with favipiravir versus the lopinavir/ritonavir group [76].

### 3.4. Ribavirin

#### 3.4.1. Efficacy

Regarding ribavirin, two randomized controlled clinical trials [77,78] and three observational studies [70,71,72] were included (Table 6).

One of the RCTs, carried out in Iran, compared the combination of ribavirin with sofosbuvir and daclastavir to the use of standard of care in hospitalized adults with moderate COVID-19 [79]. Despite not being statistically significant, positive trends in favour of the intervention group were observed, with fewer ICU admissions, lower mortality rates and lower invasive mechanical ventilation use rates. Likewise, the study did not reveal any significant differences between groups regarding length of hospital stay [77].

One of the observational studies compared the just mentioned treatments with each other, sofosbuvir/daclastavir versus ribavirin, and observed that in patients with severe COVID-19 the length of hospital stay, ICU admission rate and mortality rate were significantly lower in the group that did not receive ribavirin, thus suggesting that sofosbuvir/daclastavir are more effective than ribavirin [79].

The other RCT included in this systematic review compared the efficacy of ribavirin in combination with other drugs, specifically, 33 patients were randomized to treatment with ribavirin and IFN-α, 36 received lopinavir/ritonavir + IFN-α and 32 patients were assigned to a triple therapy of ribavirin, IFN-α and lopinavir/ritonavir [78]. The results obtained indicate that there are no statistically significant differences among the three intervention arms in terms of time to viral clearance (*p* = 0.23), time to fever resolution (p=0.55), proportion of patients with SARS-CoV-2 nucleic acid negativity at day 14 and day 28, CT-changes improvement (*p* = 0.76), length of hospital stay (*p* = 0.56) or mortality [78].

Among the studies where ribavirin was given concomitantly with other drugs, in the one by Elalfy et al. the combination treatment of ribavirin + nitazoxanide + ivermectin + zinc supplement proved to be effective in clearing SARS-CoV-2 in a shorter time than those treated with standard of care (*p* < 0.001) [80].

Finally, one of the observational studies compared the use of ribavirin in patients with severe COVID-19 with a cohort of patients who had received supportive care and found that ribavirin failed to significantly reduce mortality (*p* = 0.475) or accelerate time to viral clearance (*p* = 0.314) [81].

#### 3.4.2. Safety

Huang et al. found that the combination of ribavirin with lopinavir/ritonavir was associated with a significant increase in gastrointestinal disorders, thus suggesting that both antivirals should not be administered simultaneously in patients with COVID-19 [78].

Eslami et al., also observed that the most frequent adverse events in the ribavirin group were gastrointestinal in nature such as nausea, vomiting, diarrhoea, abdominal pain and bleeding [79].

In the study by Tong et al., ribavirin was well tolerated, with anaemia being the most frequent side effect. To this must be added, that there was no premature discontinuation of treatment as a consequence [81]. In the study by Elalfy et al. ribavirin in combination with nitazoxanide, ivermectin and zinc supplement not only proved to be more effective but was also safe and well tolerated [80].

### 3.5. Umifenovir

#### 3.5.1. Efficacy

This systematic review has included one RCT and five observational studies regarding umifenovir or better known as its trade name, arbidol (Table 7).

Starting with the Iranian RCT that enrolled 100 patients, 50 of them were assigned to a double therapy of arbidol + hydroxychloroquine, while the other half received hydroxychloroquine and lopinavir/ritonavir [82]. In this study arbidol proved to contribute significantly to clinical improvement by shortening hospitalization duration (*p* = 0.02) and by presenting a higher peripheral oxygen saturation on the seventh day of treatment (*p* = 0.02) and milder involvement of the chest-CT scan after 30 days (*p* = 0.004) [82]. 

Regarding time to defervescence, despite being numerically lower in the arbidol group, the difference was not statistically significant (*p* = 0.2). Other parameters like need of intubation (*p* = 0.6), need of mechanical ventilation (*p* = 0.6) or mortality (*p* = 0.5) did not show significant differences either [82].

In a retrospective study carried out in China, 45 patients treated with umifenovir were compared with 36 patients who had received standard therapy. Umifenovir not only failed to accelerate the length of hospital stay as well as time to viral clearance, but the clinical outcomes of this study were significantly better in the control group [83].

Another retrospective study found that arbidol compared to standard therapy could shorten time to viral clearance, as well as accelerate clinical cure by relieving fever and dry cough faster (*p* = 0.021 and *p* = 0.040, respectively). However, in terms of length of hospital stay, the differences between arbidol and standard therapy were not statistically significant [84].

Deng et al. compared in their retrospective study a cohort of patients who had received arbidol and lopinavir/ritonavir versus a control group only treated with lopinavir/ritonavir. They observed that the combination group presented higher viral negative conversion rates (*p* < 0.05) [85].

In another retrospective cohort where arbidol associated with IFN-α2b was compared with IFN-α2b monotherapy, dual therapy was not effective in terms of accelerating viral clearance (*p* = 0.057) or reducing length of hospital stay (*p* = 0.056). However, it did significantly accelerate pneumonia absorption observed through CT (*p* = 0.037) [86]. The data from this study suggest that arbidol + IFN-α2b combination therapy may be beneficial in reducing lung inflammation in mild COVID-19 patients but is powerless in shortening nucleic acid negative conversion time [86].

Arbidol given to a group of 90 patients revealed beneficial effects in comparison to 45 patients who received standard of care. These benefits were mainly observed in aspects such as higher viral clearance rates (OR = 0.23; 95% CI = 0.10–0.57; *p* = 0.002) and shorter length of hospital stay [87].

#### 3.5.2. Safety

In the included RCT, after comparing a group of patients treated with arbidol versus those allocated to lopinavir/ritonavir, it was concluded that the derived adverse events were not considerable. The most frequent ones included nausea and vomiting, which were mainly recorded in the lopinavir/ritonavir group, a result that is consistent with those observed in other studies on this drug. No fatal adverse events were recorded [82].

Similarly, in the study by Lian et al. [83], Chen et al. [84] and Gao et al. [87] no severe adverse events in patients receiving arbidol were recorded, thus suggesting that arbidol is a safe and well-tolerated drug for COVID-19 patients.

Finally, in the study by Xu et al. 18.8% of the patients treated with arbidol experienced nausea and stomach pain. However, none had to stop treatment prematurely as a consequence [86].

## 4. Discussion

Considering that it takes years to approve a new drug, in a health emergency it may be necessary to repurpose drugs that have proven to be effective in other diseases. For this reason, this systematic review has tried to gather the available evidence about five antivirals: remdesivir, ribavirin, favipiravir, umifenovir (also known as arbidol), and the dual therapy lopinavir/ritonavir, in terms of their efficacy and safety when applied to SARS-CoV-2 infected patients.

Regarding remdesivir efficacy, none of the randomized controlled clinical trials have shown statistically significant benefits in terms of mortality [46,47,49,50]. However, in general, those treated with remdesivir had a faster clinical recovery compared to their respective control groups [46,48,49].

When it comes to the question of how long remdesivir should be given, there are discrepancies between studies. In the study by Spinner et al. remdesivir therapy for 5 days was more likely to have a better clinical status on day 11 compared to standard of care [46].

Parallelly, Goldman et al. compared a 5-day regimen of remdesivir versus a 10-day course and despite noticing a trend toward better outcomes in patients receiving it for 5 days, the differences were not statistically significant [47]. These results have been compared with those of a meta-analysis published online in March 2021, where five of the RCTs included in this systematic review are analysed [88].

On the one hand, this meta-analysis reveals that those who received a 5-day remdesivir therapy had greater clinical improvement compared to those in the control group (OR = 1.68; 95% CI = 1.18–2.40). On the other hand, no statistically significant differences were observed between those with a 10-day remdesivir regimen and those receiving placebo (OR = 1.23; 95% CI = 0.90–1.68) [88].

Contrary to the results recorded in the RCTs, several of the included observational studies, do detect a significant reduction in mortality in remdesivir group [51,52]. 

However, it must be taken into account that the lack of randomization, the small sample size in the case of Pasquini et al. [51] or the use of other medications as standard therapy in the study by Olender et al. [52], may have had an impact on the results.

Regarding safety, no significant association has been found between an increased risk of suffering an adverse event and the use of remdesivir [46,48,49,53].

Large randomized controlled clinical trials on the use of lopinavir/ritonavir applied to COVID-19 patients, have revealed a lack of efficacy in terms of clinical improvement, mortality reduction, viral clearance, length of hospital stay, oxygenation-free days and mechanical ventilation-free days [50,55,56,58].

These results are consistent with those of a meta-analysis published online in April 2021 where the differences between lopinavir/ritonavir group and those receiving standard therapy were not statistically significant in terms of mortality, viral clearance (RR = 1.06; 95% CI = 0.85–1.31) and radiological improvement (RR = 0.81; 95% CI = 0.62–1.05) [89].

The majority of the observational studies included in this systematic review coincide with the results proposed by the RCTs and neither did they find benefits regarding the use of lopinavir/ritonavir compared to standard of care [59,60,61,63,64,67]. In fact, in three of them standard therapy was even more effective [60,61,64].

Other authors have compared the efficacy of lopinavir/ritonavir administered simultaneously with other drugs such as IFN-ß1a [58] or hydroxychloroquine [65,67], without detecting any significant differences between these combination therapies and their respective control groups.

Regarding safety, gastrointestinal adverse events like nausea, vomiting and diarrhoea have been more common in those who received lopinavir/ritonavir [55,57,65].

While it is true that most of the studies included in this review recorded mild and self-limited adverse events, in some studies like the one by Grimaldi et al. there was a higher incidence of acute renal injury and need for renal replacement therapy in those receiving lopinavir/ritonavir, thus raising doubts about its safety profile [60]. 

Likewise, lopinavir/ritonavir proved to be inadequate to treat paediatric population since the number of adverse events was significantly higher in this group compared to those who received standard therapy [64].

Treatment with favipiravir has shown efficacy in accelerating clinical cure, specifically time to defervescence [69,71,73]. However, only one study has registered a higher viral clearance compared to standard of care [73].

It should be noted that the benefits recorded in the study by Balykova et al. regarding the use of favipiravir versus standard of care are difficult to quantify since the standard therapy consisted of various combinations of drugs, mainly hydroxychloroquine, azithromycin and lopinavir/ritonavir [71].

In a meta-analysis published in September 2020, several of the studies included in this systematic review were analysed. The group of patients treated with favipiravir showed a significant clinical improvement by day 14 (RR = 1.29, 95% CI = 1.08–1.54) compared to the control group. In contrast, viral clearance by 14 day (RR = 1.06; 95% CI = 0.84–1.33) and need for oxygen or non-invasive mechanical ventilation (OR = 0.76, 95% CI = 0.42–1.39) did not show statistically significant differences between groups [90].

The combination of favipiravir with tocilizumab seems to have promising results in terms of reducing lung parenchyma inflammation, as well as in recovering clinical symptoms compared to favipiravir monotherapy [74]. Finally, two observational studies revealed that treatment with favipiravir was superior to lopinavir/ritonavir [75,76].

In terms of safety, patients treated with favipiravir experienced fewer adverse events than their respective control groups, being the most common symptom hyperuricemia. The results obtained in the just mentioned meta-analysis, revealed no significant differences between favipiravir and control group (OR = 0.69, 95% CI = 0.13–3.57) [90].

Ribavirin has not proven to be effective in reducing mortality, time to viral clearance, length of hospital stay, or in alleviating symptoms in any of the studies included in this review [77,78,79,81].

Some of the studies assessed the efficacy of ribavirin combined with other drugs [77,78,80], but only in one of them ribavirin appeared to be superior to standard of care in suppressing viral shedding [80], suggesting that sofosbuvir/daclastavir combination is more effective than ribavirin. In this regard, in an open-label RCT conducted in patients with mild to moderate COVID-19, although the combination of sofosbuvir and daclatasvir accelerated the time to clinical response, it failed to improve the length of hospital stay or 14-day mortality [91]. In a meta-analysis including two randomized and one non-randomized clinical trials and conducted among patients with moderate to severe COVID-19, those treated with sofosbuvir/daclatasvir did not only show a greater clinical recovery compared to their respective control groups at 14 days after randomization, but also improved time to clinical recovery and had a significantly lower combined risk of mortality [92]. However, while in another study, sofosbuvir/daclatasvir did not show a significant efficacy in terms of hospital discharge and survival rates [93] the number of patients with dyspnoea and fatigue was significantly lower after one month [94]. 

In terms of safety ribavirin was well tolerated, however, its concomitant use with lopinavir/ritonavir seems to potentiate gastrointestinal disorders, therefore this combination is not recommended in patients with COVID-19 [78].

We have only been able to include one RCT on arbidol in this systematic review. Here, the association of arbidol with hydroxychloroquine significantly reduced the length of hospital stay, as well as improved radiological lung parenchyma changes compared to the group treated with lopinavir/ritonavir [82].

Likewise, one of the included observational studies also verified the superiority of arbidol against lopinavir/ritonavir and registered significantly higher viral clearance in the group treated with arbidol [85]. This apparent superiority of arbidol over lopinavir/ritonavir is consistent with the results observed in the study by Zhu et al. [66].

Finally, three studies compared the efficacy of arbidol versus standard therapy [83,84,87]. Two of them showed results in favour of arbidol regarding viral clearance [84,87] and only one study observed a significant shorter length of hospital stay [87].

None of the studies detected serious adverse events leading to discontinuation of treatment because of the use of arbidol. 

A meta-analysis published in January 2021 supports some of the results just presented, such as the safety of arbidol (RR for adverse events = 1.29; 95% CI = 0.57–2.92) or the lack of efficacy of arbidol in reducing the length of hospital stay [95].

Some of the antivirals of the current paper have either been administered concomitantly or compared with hydroxychloroquine. This drug is a 4-aminoquinoline compound that has been used as an antimalarial for years [96]. Due to its low cost and oral administration, it has been considered as a prospective repurposed candidate drug to treat COVID-19 patients [96]. However, to date the use of hydroxychloroquine alone has not shown benefits in treating hospitalized COVID-19 patients [96,97].

In fact, given its lack of benefit in terms of mortality, the hydroxychloroquine arms were discontinued in both SOLIDARITY [98] and RECOVERY [99,100] clinical trials. Moreover, in June 2020, the FDA revoked the authorization for the emergency use of hydroxychloroquine in COVID-19 patients [101].

These findings have subsequently been confirmed by several meta-analyses. In one published in September 2020, those treated with hydroxychloroquine failed to achieve a significant reduction in mortality (RR = 0.98; 95% CI = 0.66–1.46) as well as to shorten time to defervescence compared to those treated with standard of care [102]. The use of hydroxychloroquine was also associated with an increased risk of electrocardiogram abnormalities and arrhythmias (RR = 1.46; 95% CI = 1.04–2.06) [102]. In another meta-analyses published in April 2021 hydroxychloroquine therapy was associated with increased mortality in COVID-19 patients compared to placebo or standard of care [103]. In short, due to its lack of efficacy and its potential risks, the use of hydroxychloroquine alone in COVID-19 patients is not recommended [97].

Once summarized evidence on the efficacy and safety of some antivirals that have proven to be effective, there is no doubt that the SARS-CoV-2 pandemic implies a major threat to human health. However, while it is essential to find a vaccine to immunize the population, this is a long-term solution. 

In the meantime, we must continue to invest in and support pharmacological research. Firstly, to be able to treat already infected patients. Secondly, to ensure that in regions where access to vaccines is limited, there are means to counteract this disease. Thirdly, since it is yet unclear whether the authorized vaccines are effective against the new viral strains of concern, which are the result of mutations accumulation [104].

As a reflection, given the similarity of this new virus to the coronaviruses responsible for the SARS and MERS epidemics in 2002 and 2012 respectively, we could think that, almost a decade later, effective antivirals would be available to treat them. However, this is not the case. This leads us to believe that, by affecting only a limited geographical region, once the incidence started to decrease and the disease was controlled locally, it lost the interest of large institutions to continue researching on a suitable pharmacological treatment. Hence, it is very important to keep researching, even when effective vaccines are already available or the incidence is starting to decline. New variants of the virus may emerge in the future or a new wave of cases may arise, for which having an effective drug would be very helpful.

### Limitations

The main limitations found in the studies included in this systematic review are the following. On the one hand, most of them had an open design, so knowing the drug being administered may have influenced the clinical outcomes. On the other hand, many of the studies did not collect detailed information about adverse events or reasons for abandoning treatment. Therefore, the conclusions obtained in terms of safety are reduced, which limits their interpretation.

The absence of placebo as a comparative method in most of the included studies makes it difficult to determine whether the observed results are due to the evolution of the curative process of the disease itself, to the antiviral treatment used, or to a combination of both. Another limitation would be the small sample size used in some of the studies.

In the studies where patients with COVID-19 of mild to moderate severity were recruited, it was more difficult to assess the symptoms, thus contributing to a possible underestimation of potential clinical benefits of the antivirals applied. That is why, the results of these studies cannot be extrapolated to severe or critically ill COVID-19 patients.

Many of the antiviral agents were used in combination with other medications, implying that the observed results cannot be exclusively attributed to the antiviral treatment.

Finally, it should be noted that even though systematic reviews constitute the “gold standard” when it comes to synthesizing the evidence to answer a scientific question, these types of articles require a great investment of time, which makes their information quickly obsolete, especially in an emergency health situation like the current one, in which new studies are constantly being published.

## 5. Conclusions

- Remdesivir may help accelerate clinical improvement in hospitalized COVID-19 patients, but it is not effective in reducing mortality. Regarding the duration of the treatment, a 5-day course seems to be sufficient. In terms of safety, this drug has proven to be as tolerable as placebo or other drugs that it has been compared to.
- Lopinavir/ritonavir has not proven to be an effective treatment for patients with SARS-CoV-2 infection in improving clinical status, reducing mortality, or increasing viral clearance. In fact, it has been associated with a higher risk of gastrointestinal adverse events.
- Treatment with favipiravir is associated with significant clinical improvement, mainly in reducing time to fever resolution, and is also safe and well-tolerated. Likewise, the combination of favipiravir with tocilizumab seems to have promising results in terms of reducing lung parenchyma inflammation and symptom resolution. However, the use of favipiravir has failed to increase viral clearance.
- Ribavirin has not proven to be an effective treatment for COVID-19 patients, and its concomitant use with lopinavir/ritonavir is not recommended, due to an increase in the risk of adverse events.
- Arbidol has proven to be a safe and well-tolerated treatment for COVID-19 patients. However, it is not associated with a significant reduction in the length of hospital stay. Due to the small number of studies included in this systematic review regarding arbidol, we do not have sufficient evidence to support the use of this drug in patients with COVID-19.

## Figures and Tables

**Table 1:** Therapies studied for COVID-19 treatment in clinical trials. Abbreviations: ACE-2: angiotensin-converting enzyme 2, CYP450: cytochrome P450, HIV-1: human immunodeficiency virus type 1, IL-6: interleukin 6, RdRp: RNA-dependent RNA polymerase, RNA: ribonucleic acid, SARS-CoV-2: coronavirus type 2 that causes severe acute respiratory syndrome, TMPRSS2: transmembrane serine protease 2.

Category	Group	Drug	Supposed Mechanism of Action
**Antivirals**	Cell membrane fusion inhibitors	Umifenovir (Arbidol) [28]	Blocks the entry and intracellular vesicular traffic
	RNA polymerase inhibitors / RNA synthesis inhibitors	Remdesivir (GS-5734) [29]	Nucleotide analog (Adenosine), prodrug, RNA-dependent RNA polymerase (RdRp) inhibitor
		Favipiravir [30,31,32]	Nucleotide precursor analog, oral drug, inhibits the viral RNA dependent RNA polymerase (RdRp) (its triphosphoribosylated form, is recognized as a substrate RdRp) acting as a chain terminator and inhibiting the viral polymerase activity
		Ribavirin [33]	Nucleoside analogue, RNA-dependent RNA polymerase (RdRp) inhibitor
	Viral protease inhibitors	Lopinavir/Ritonavir [23]	Lopinavir (HIV-1 aspartate protease inhibitor); Ritonavir (CYP450 inhibitor)
**Antimalarials**	Aminoquinolines	Chloroquine/Hydroxychloroquine [34]	Increases endosomal pH and hinders the interaction of the virus with the ACE-2 receptor on the cell surface.
	Serine protease inhibitors	Camostat Mesylate [30]	Blocks the entry of SARS-CoV-2 into the host cell by inhibiting TMPRSS2
**Immunotherapy**	IL-6 inhibitors [35]	Tocilizumab	Blocks soluble IL-6 receptors attached to the cell membrane.
		Sarilumab	Blocks soluble IL-6 receptors attached to the cell membrane
	Convalescent plasma or hyperimmune immunoglobulins [33]		Treatment by plasma or purified monoclonal antibodies produced in patients already recovered from COVID-19
**Corticosteroids**		Dexamethasone Hydrocortisone [33]	Reduce the host’s inflammatory response in the lungs

**Table 2:** Inclusion criteria based on PICO algorithm. ICU: intensive care unit.

**Patient (P)**	We included individuals with a confirmed diagnosis of COVID-19, with no restrictions on age, sex, ethnicity, or severity of the disease.
**Intervention (I)**	We included the following antiviral treatments: remdesivir, lopinavir/ritonavir, favipiravir, umifenovir, or ribavirin. Co-interventions with other antivirals were allowed but must be comparable between intervention groups.
**Comparison (C)**	We included the following comparisons for studies with a control arm: placebo, standard care, another dosage regimen, any other drug treatment (including but not limited to antivirals)
**Outcome (O)**	We included studies that evaluate efficacy of interventions being this estimated by mortality rate, improvement of clinical symptoms, need for mechanical ventilation, length of hospital stay, number of patients admitted to ICU, length of stay on the ICU, rate of viral clearance or time to viral clearance. We also included studies that assess safety outcomes as measured by proportion of mild, moderate, or severe adverse events. Secondary outcomes include need to discontinue treatment because of adverse events, dosage regimen, and duration of antiviral treatment.

**Table 3:** Characteristics of included studies on remdesivir. Abbreviations: CG: control group, IG: intervention group, IG1: intervention group 1, IG2: intervention group 2, IG3: intervention group 3, MV: mechanical ventilation [IQR]: interquartile range, No: number of patients, RCT: randomized controlled trial, SD: standard deviation, WHO: World Health Organization, ♂: male.

Reference	Study Design/Population of Study	No. of Participants	Median Age, [IQR], Years	Sex ♂
**Remdesivir vs. Standard of care**				
Spinner C., et al., 2020 [46]	RCT (open-label, phase III, multicentre)/Adults with moderate COVID-19	IG_1_: n = 197 IG_2_: n = 199 CG: n = 200	IG_1_: 56 [45–66] IG_2_: 58 [48–66] CG: 57 [45–66]	IG_1_: 61% IG_2_: 60% CG: 63%
**Remdesivir for 5 days vs. 10 days**				
Goldman J., et al., 2020 [47]	RCT (open-label, phase III, multicentre)/Patients with severe COVID-19	IG: n = 200; CG: n = 197	IG: 61 [50–69] CG: 62 [50–71]	IG: 60% CG: 68%
**Remdesivir vs. Placebo**				
Beigel J., et al., 2020 [48]	RCT (double blind, multicentre, placebo-controlled)/Adults with moderate or severe COVID-19	IG: n = 541 CG: n = 521	Mean age, ± SD: IG: 58.6 ± 14.6 CG: 59.2 ± 15.4	IG: 65.1% CG: 63.7%
**Remdesivir vs. Placebo**				
Wang Y., et al., 2020 [49]	RCT (double blind, multicentre, placebo-controlled)/Adults with severe COVID-19	IG: n = 158 CG: n = 78	IG: 66 [57–73] CG: 64 [53–70]	IG: 56% CG: 65%
**Remdesivir vs. Standard of care**				
Pan et al., 2021 [50]	RCT (open-label, international, multicentre) WHO Solidarity trial consortium/Adults with COVID-19	IG: n = 2743 CG: n = 2708	Age 50–69 years: IG: 46.7% CG: 47.5%	IG: 62.2% CG: 63.7%
**Remdesivir vs. Standard of care**				
Pasquini Z., et al., 2020 [51]	Observational and retrospective study/Critically ill patients under MV with confirmed COVID-19 and admitted to the intensive care unit	IG: n = 25 CG: n = 26	IG: 64 [57–75] CG: 70 [63.3–76]	IG: 92% CG: 92.3%
**Remdesivir vs. Standard of care**				
Olender S., et al., 2020 [52]	IG: data from a phase III prospective, randomized *RDV* trial; CG: data from a longitudinal retrospective cohort/Adults with severe COVID-19	IG: n = 312 CG: n = 818	Age 40–64 years IG: 50% CG: 50%	IG: 59% CG: 59%
R**emdesivir vs. Supportive care**				
Kalligeros M., et al., 2020 [53]	Observational, retrospective study/Adults with severe COVID-19	GI: n = 99 GC: n = 125	IG: 58 [50–68] CG: 60 [50–68]	IG: 69.7% CG: 64.8%
**(Barticinib + Remdesivir) vs. Remdesivir**				
Kalil P., et al., 2020 [54]	Double blind RCT (placebo-controlled)/Hospitalized adults with COVID-19	IG: n = 515 CG: n = 518	Mean age, ± SD: IG: 55 ± 15.4: CG: 55.8 ± 16	IG: 61.9% CG: 64.3%

**Table 4:** Characteristics of included studies on lopinavir/ritonavir. Abbreviations: CG: control group, ICU: intensive care unit, IG: intervention group, IG1: intervention group 1, IG2: intervention group 2, IG3: intervention group 3, [IQR]: interquartile range, N/A: not available, No: number of patients, RCT: randomized controlled trial, SD: standard deviation, WHO: World Health Organization, ♂: male.

Reference	Study Design /Population of Study	No. of Participants	Median Age [IQR], Years	Sex ♂
**Lopinavir/Ritonavir vs. Standard of care**				
Cao B., et al., 2020 [55]	RCT (open-label)/Adults with severe COVID-19	IG: n = 99 CG: n = 100	IG: 58 [50–68] CG: 58 [48–68]	IG: 61.6% CG: 59.0%
**Lopinavir/Ritonavir vs. Standard of care**				
Horby, P., et al., 2020 [56]	RCT (open-label) RECOVERY/Patients with COVID-19	IG: n = 1616 CG: n = 3424	Mean age, ± SD: IG: 66 ± 16 CG: 66.4 ± 15.8	IG: 60% CG: 61%
**(Lopinavir/Ritonavir + Ribavarin + IFNβ1b) vs. Lopinavir/Ritonavir**				
Hung I., et al., 2020 [57]	RCT (phase II, open-label, multicentre)/Adults with mild to moderate COVID-19	IG: n = 86 CG: n = 41	IG: 51 [31.0–61.3] CG: 52 [33.5–62.5]	IG: 52% CG: 56%
**Lopinavir/Ritonavir vs. Standard of care**				
Pan et al., 2021 [50]	RCT (open-label, international, multicentre) WHO Solidarity trial consortium/Adults with COVID-19	IG: n = 1399 CG: n = 1372	Age 50–69: IG: 42.67% CG: 43.44%	IG: 60.83% CG: 58.45%
**Lopinavir/Ritonavir vs. Standard of care // (Lopinavir/Ritonavir + IFN-β1a) vs Standard of care**				
Ader F., et al., 2021 [58]	RCT (open-label, multicentre, phase III) DisCoVery trial/Patients with moderate to severe COVID-19	IG_1_: n = 145 IG_2_: n = 145 CG: n = 148	IG_1_: 63 [55–71] IG_2_: 64 [53–71] CG: 62 [52–71]	IG_1_: 73.1% IG_2_: 71% GC: 70.9%
**Lopinavir/Ritonavir vs. Standard of care**				
Gao, G., et al., 2020 [59]	Observational, retrospective study/Adults with non-severe COVID-19	IG_1_: n = 51 CG: n = 59	IG_1_: 33 [27–41] CG: 30 [23–45]	IG_1_: 58.8% CG: 50.8%
**Lopinavir/Ritonavir vs. Standard of care**				
Grimaldi D., et al., 2020 [60]	Observational multicentre cohort study/Adults with moderate to severe COVID-19	IG_1_: n = 57 CG: n = 85	Mean age, ± SD: IG_1_: 63 ± 12 CG: 63 ± 11	IG_1_: 80% CG: 75%
**Lopinavir/Ritonavir vs. Standard of care**				
Choi M., et al., 2020 [61]	Observational retrospective cohort study/Adults with mild to moderate COVID-19	IG_1_: n = 1407 CG: n = 1407	Mean age, ± SD: IG_1_: 45.9 ± 15.6 CG: 45.9 ± 15.6	IG_1_: 38.59% CG: 38.59%
**Lopinavir/Ritonavir vs. Standard of care**				
Ye X, et al., 2020 [62]	Observational, retrospective study/Patients with COVID-19	IG: n = 42 CG: n = 5	N/A	IG: 50% CG: 20%
**Lopinavir/Ritonavir vs. Standard of care**				
Lecronier M., et al., 2020 [63]	Observational, retrospective study/Patients with severe COVID-19 requiring ICU admission	IG_1_: n = 20 IG_2_: n = 38 CG: n = 22	IG_1_: 55 [49–61] IG_2_: 59 [53–66] CG: 63 [54–70]	IG_1_: 75% IG_2_: 82% CG: 82%
**Lopinavir/Ritonavir vs. Standard of care**				
Lu J., et al., 2020 [64]	Observational, retrospective, multicentre study/Paediatric patients with mild COVID-19	IG: n=23 CG: n=92	IG: 8.66 [2.44–11.9] CG: 8.85 [2.00–11.6]	IG: 56.52% CG: 56.52%
**Lopinavir/Ritonavir + Hydroxychloroquine: Early treatment (<5 days from symptom onset) vs. Late treatment (>5 days from symptom onset)**				
Giacomelli A., et al., 2020 [65]	Retrospective cohort study/Adults with COVID-19	IG: n = 43 CG: n = 129	IG: 64.9 [55–78] CG: 61.7 [50.2–72.3]	IG: 67.4% CG: 73.6%
**Lopinavir/Ritonavir vs. Arbidol**				
Zhu, Z., et al., 2020 [66]	Observational, retrospective study/Adults with COVID-19	IG: n = 34 CG: n = 16	IG: 40.5 [34.8–52.3] CG: 26.5 [23.3–52.5]	IG: 58.8% CG: 37.5%
**Lopinavir/Ritonavir vs. Standard of care**				
Vernaz N., et al., 2020 [67]	Observational, retrospective, single centre, cohort study/Patients with COVID-19	IG_1_: n = 93 IG_2_: n = 83 IG_3_: n = 158 CG: n = 506	Mean age, ± SD: IG_1_: 66.14 ± 15.77 IG_2_: 63.4 ± 17.4 IG_3_: 62.15 ± 14.77 CG: 70.75 ± 20.01	IG1: 59.1% IG_2_: 55.4% IG_3_: 36.1% CG: 43.9%
**Lopinavir/Ritonavir vs. Standard therapy**				
Yan D., et al., 2020 [68]	Observational, retrospective study/Non critically ill COVID-19 patients	IG: n = 78 CG: n = 42	IG: 50 [34–61] CG: 57 [36.5–66]	IG: 44.9% CG: 45.2%

**Table 5:** Characteristics of included studies on favipiravir. Abbreviations: CG: comparison group, ICU: intensive care unit, IFN-α1b: Interferon α1b, IG: intervention group, IG1: intervention group 1, IG2: intervention group 2, [IQR]: interquartile range, N/A: not available, No: number of patients, RCT: randomized controlled trial, SD: standard deviation, ♂: male.

Reference	Study Design/Population of Study	No. of Participants	Median Age, [IQR], Years	Sex ♂
**Favipiravir early vs. late administration**				
Doi Y., et al., 2020 [69]	RCT (open-label, prospective, multicentre)/Patients with asymptomatic or mild COVID-19	IG: n = 44 CG: n = 45	IG: 48 [34.5–68] CG: 51 [39.5-62]	IG: 52.3% CG: 70.5%
**Favipiravir vs. Supportive care**				
Udwadia Z., et al., 2020 [70]	RCT (open-label, phase III, multicentre)/Adults with mild to moderate COVID-19	IG: n = 72 CG: n = 75	Mean age, ± SD: IG: 43.6 ± 12.2 CG: 43 ± 11.2	IG: 70.8% CG: 76%
**Favipiravir vs. Standard of care**				
Balykova L., et al., 2020 [71]	RCT (open-label, multicentre)/Adults with moderate COVID-19 and presence of bilateral pneumonia	IG: n = 17 CG: n = 22	Mean age, ± SD: IG: 47.12 ± 2.26 CG: 47.5 ± 1.99	N/A
**Favipiravir vs. Chloroquine**				
Dabbous H., et al., 2020 [72]	RCT (open-label, phase II/III, multicentre)/Adults with mild to moderate COVID-19	IG: n = 44 CG: n =48	Mean age, ± SD: IG: 34.86 ± 15.9 CG: 36.15 ± 17.7	IG: 45.5% CG: 52.1%
**Favipiravir vs. Standard of care**				
Ivashchenko, A., et al., 2020 [73]	RCT (open-label, phase II/III, multicentre)/Adults with moderate COVID-19	IG_1_: n = 20 IG_2_: n = 20 CG: n = 20	N/A	N/A
**(Favipiravir + Tocilizumab) vs. Favipiravir**				
Zhao H., et al., 2020 [74]	RCT (multicentre)/Adults with COVID-19	IG: n = 14 CG: n = 7	IG: 75 [34–81] CG: 70 [45–89]	IG: 42.9% CG: 71.4%
**(Favipiravir + IFN-α1b) vs. (Lopinavir/Ritonavir + IFN-α1b)**				
Cai Q., et al., 2020 [75]	Comparative controlled study (non-randomized, open-label)/Patients with confirmed COVID-19	IG: n = 35 CG: n = 45	IG: 43 [35.5–59] CG: 49 [36–61]	IG: 40% CG: 46.7%
**(Favipiravir + Hydroxychloroquine) vs. (Lopinavir/Ritonavir + Hydroxychloroquine)**				
Kocayiğit H., et al., 2020 [76]	Observational retrospective study/Critically ill patients with COVID-19 (admitted to ICU)	IG: n = 65 CG: n = 42	Mean age, ± SD: IG: 69.8 ± 12.6 CG: 70.6 ± 12.7	IG: 58.5% CG: 64.3%

**Table 6:** Characteristics of included studies on ribavirin. Abbreviations: CG: comparison group, IFN-α: interferon alpha, IG: intervention group, IG1: intervention group 1, IG2: intervention group 2, IG3: intervention group 3, [IQR]: interquartile range, No: number of patients, RCT: randomized controlled trial, SD: standard deviation, ♂: male.

Reference	Study Design/Population of Study	No. of Participants	Median Age, [IQR], Years:	Sex ♂
**(Ribavirin + Sofosbuvir + Daclatasvir) vs. Standard therapy**				
Abbaspour Kasgari H., et al., 2020 [77]	RCT (single centre)/Adults with moderate COVID-19	IG: n = 24 CG: n = 24	IG: 45 [38–69] CG: 60 [47.5–68.5]	IG: 46% CG: 29%
**(Ribavirin + IFN-α) vs. (Lopinavir/Ritonavir + IFN-α) vs (Ribavirin + Lopinavir/Ritonavir + IFN-α)**				
Huang Y., et al., 2020 [78]	RCT (open-label, single centre, prospective)/Adults with mild to moderate COVID-19	IG_1_: n = 33 IG_2_: n = 36 IG_3_: n = 32	Mean age, ± SD: IG_1_: 40.3 ± 12.5 IG_2_: 43.3 ± 10.4 IG_3_: 43.8 ± 11.7	IG_1_: 55% IG_2_: 53% IG_3_: 28%
**Sofosbuvir/Daclastavir vs. Ribavirin**				
Eslami G., et al., 2020 [79]	Open-label, parallel trial, prospective, pseudorandom allocation/Adults with severe COVID-19	IG: n = 35 CG: n = 27	IG: 62 [47–69] CG: 60 [43–73]	IG: 49% CG: 52%
**(Ribavirin + Nitazoxanide + Ivermectin + Zinc supplement) vs. Standard of care**				
Elalfy H., et al., 2020 [80]	Controlled clinical trial (non-randomized, Phase I)/Adults with mild to moderate COVID-19	IG: n = 62 CG: n = 51	Mean age, ± SD: IG: 37.9 ± 11.9 CG: 37.5 ± 10.9	IG: 48.4%; CG: 43.1%
**Ribavirin vs. Supportive care**				
Tong S., et al., 2020 [81]	Observational, retrospective, single centre cohort study/Patients with severe COVID-19	IG: n = 44 CG: n = 71	Mean age, ± SD: IG: 54.6 ± 13.3 CG: 55.1 ± 16.2	IG: 43.2%; CG: 60.6%

**Table 7:** Characteristics of included studies on umifenovir. Abbreviations: CG: comparison group, ICU: intensive care unit, IFN-α2b: Interferon α2b, IG: intervention group, IMV: invasive mechanical ventilation, [IQR]: interquartile range, N/A: not available, No: number of patients, RCT: randomized controlled trial, SD: standard deviation, ♂: male.

Reference	Study Design/Population of Study	No. of Participants	Median Age, [IQR], Years	Sex ♂
**Arbidol vs. Lopinavir/Ritonavir**				
Nojomi M., et al., 2020 [82]	RCT (open-label)/Adults with COVID-19	IG: n = 50 CG: n = 50	Mean age, ± SD: IG: 56.6 ± 17.8 CG: 56.2 ± 14.8	IG: 66% CG: 54%
**Umifenovir vs. Standard of care**				
Lian N., et al., 2020 [83]	Observational, retrospective study (single centre)/Adults with COVID-19 in a non-ICU	IG: n = 45 CG: n = 36	IG: 58 [50–66] CG: 63 [49–66]	IG: 62% CG: 47%
**Arbidol vs. Standard of care**				
Chen W., et al., 2020 [84]	Observational, retrospective study/Adults with COVID-19	IG: n = 42 CG: n = 20	IG: N/A CG: N/A	IG: 57.1% CG: 50%
**(Arbidol + Lopinavir/Ritonavir) vs. Lopinavir/Ritonavir**				
Deng L., et al., 2020 [85]	Observational retrospective cohort study (single centre)/Adults with COVID-19 without IMV	IG: n = 16 CG: n = 17	Mean age, ± SD: IG: 41.8 ± 14.08 CG: 47.25 ± 17.25	IG: 43.8% CG: 58.8%
**(Arbidol + IFN-α2b) vs. IFN-α2b**				
Xu P., et al., 2020 [86]	Retrospective, multicentre cohort study/Adults with COVID-19 without IMV	IG: n = 71 CG: n = 70	Median age, [range] IG: 50.9 [24–75] CG: 53.2 [26–83]	IG: 57.7% CG: 47.1%
**Arbidol vs. Standard therapy**				
Gao W., et al., 2020 [87]	Observational, retrospective cohort study/Patients with COVID-19	IG: n = 90 CG: n = 45	IG: 48 [36–56] CG: 51 [40–61]	IG: 57.8% CG: 48.9%

## Data Availability

Not applicable.
